# Dataset of *De novo* leaf, salicylic induced leaf and flower transcriptome profiling of *Datura metel* plant

**DOI:** 10.1016/j.dib.2023.109737

**Published:** 2023-10-29

**Authors:** Madhavi Hewadikaram, S.D.N.K. Bathige, Veranja Karunaratne

**Affiliations:** a NSBM Green University, Pitipana, Homagama, Sri Lanka; b Sri Lanka Institute of Nanotechnology, Nanotechnology and Science Park, Mahenwatte, Pitipana, Homagama 10206, Sri Lanka; c SLTC Research University, Padukka, Sri Lanka

**Keywords:** Transcriptomics, *De novo* sequencing, Secondary metabolites, Gene annotations, Leaf, *Datura metel*

## Abstract

*Datura metel* L (thorn's apple) is a popular plant belonging to the family Solanaceae, growing all around the year in humid and warm climates. The importance of *D. metel* as a medicinal marvel is due to secondary metabolites within various parts of the plant, which serve different therapeutic functions. The whole plant is considered a narcotic, anodyne, and antispasmodic, while the leaves, bark, and seeds are also separately used in extractions. The biological potency of the plant has been used in traditional medicine for over a century. Currently, plant parts are used as a rich source in pharmaceutical manufacturing of secondary metabolites such as flavonoids, saponins, alkaloids, steroids, tannins, and withanaloids. *D. metel* has proven advanced functions of antiviral effects, antibacterial and antifungal effects, anti-inflammatory, analgesic, antipyretic, hepatoprotective, nephroprotective effect, anticancer, and to treat chronic cardiovascular diseases, diabetes, and neurological ailments. This is the first report on transcriptome assembly for this plant. The raw RNA sequencing data for leaf, salicylic-induced leaf, and flower are available at the NCBI Sequence Read Archive (SRA) under the Bioproject access PRJNA838784. The raw RNA sequencing data that is currently accessible can be utilized to conduct differential gene expression investigations pertaining to various secondary metabolite pathways and diverse tissues, as well as for the research of gene expression related to stress induced by salicylic acid in leaf tissues of the plant. Gene functions can be evaluated and mostly utilized for gene clustering data analysis, gene characterizations, and the identification of genes involved in linked biological pathways in plant studies.

Specifications Table

**Table:** 

Subject	Omics: Transcriptomics
Specific subject area	*De novo* sequencing and transcriptomic analysis of important medicinal plant tissues
Data format	Raw
Type of data	Table
Data collection	A salicylic acid (SA) concentration of 5 mM was sprayed on the third leaf every 12 h over a span of 36 h. Water was sprayed as the control for another three plants. Three hours following the final treatment, SA sprayed leaves, normal leaves, and flower tissues were excised (two biological repeats from the tissue type) and immersed in an RNA stabilization reagent, RNAlater (Cat 76,104, Qiagen, Germany), to facilitate RNA isolation and subsequent transcriptome analysis. RNA extraction was executed using a Qiagen RNeasy plant mini kit, in adherence to the manufacturer's instructions. Quantitative and qualitative RNA assessment employed a NanoDrop-1000 spectrophotometer (Thermo Fisher Scientific Inc., Waltham, USA). The RNA purity was determined through absorbance measurements at 260 and 280 nm (A260/280) and 260/230 nm (A260/230), with ratios between 1.8 and 2.0 indicating optimal purity. RNA integrity was evaluated using an Agilent 2100 bioanalyzer, with a minimum RIN of 7.5 established as the threshold for our samples. **RNA sequencing** RNA sequencing and library preparation were done at Macrogen Korea. There, the ribosomal RNA (rRNA) was briefly removed from Ribo-Zero rRNA removal beads using TruSeq Stranded Total RNA with Ribo-Zero Plant (Illumina). Following purification, the RNA is fragmented into small pieces using divalent cations under elevated temperatures. The cleaved RNA fragments are copied into the first-strand cDNA using reverse transcriptase and random primers, followed by second-strand cDNA synthesis using DNA polymerase I and RNase H enzyme. The products were purified and enriched in a PCR to create the final cDNA library. The QC of the amplified library was determined using a high-sensitivity bioanalyzer chip (Agilent Technologies Inc., Santa Clara, CA). Then the libraries were sequenced on Illumina NovaSeq 6000, and 100 base-pair end sequencing was conducted. Short read data in FASTQ file format were produced and the short reads that did not pass Illumina standard quality filter were eliminated. The process yielded clean reads from the mRNA pool isolated from *D. metel* tissues of leaves, induced leaves, and flower. **RNA sequencing analysis (RNA-Seq analysis)** Raw reads were filtered to exclude reads containing adapter and low-quality sequences using Trimmomatic software. The N20, N30, and N50 values, the GC content, and the clean data sequence duplication level were calculated. *De novo* assembly of each transcriptome and a common transcriptome of leaf, induced leaf, and flower buds were performed with Trinity 2.8.4 software. The default Kmer size of 25 was set for the *de novo* transcriptome assembly of the flowers and leaves. The length of the assembled unigenes used for further study was ≥500 bp. All downstream analyses were based on high-quality clean data. CD-HIT-EST (version 4.6.3) analysis was performed by selecting the longest isoform, and other contigs were merged according to a similarity criterion of 90 %. This would eliminate the redundancy of the FASTA sequences generated from assembly. BUSCO assessment was conducted to find the completeness of transcriptome assembly under viridiplantea (Kingdom) lineage and cut-off E value of 1.03E^−3^.
Data source location	The greenhouse facility at Sri Lanka Institute of Nano Technology, Sri Lanka
Data accessibility	Repository name: *Datura metel* Raw sequence reads. Data identification number: **PRJNA838784** Direct URL to data: https://www.ncbi.nlm.nih.gov/bioproject/PRJNA838784/ Under Biosamples Flower Raw RNAseq data are deposited in **SRX17976631** Link: https://www.ncbi.nlm.nih.gov/sra/SRX17976631[*accn*] RNA-seq of Salicylic induced leaf tissue analysis in **SRX17976630** Link: https://www.ncbi.nlm.nih.gov/sra/SRX17976630[*accn*] RNA-seq for leaf normal tissue analysis in **SRX17976629** Link: https://www.ncbi.nlm.nih.gov/sra/SRX17976629[accn]

## 1 Value of the Data

• The integration of transcriptomics and metabolomics knowledge, along with their applications, will provide a more comprehensive path for gathering all information related to plant metabolomics. This approach is critical for identifying missing genes and transcription factors that regulate the production of secondary metabolites within their respective biosynthesis pathways. A deep understanding of secondary metabolite biosynthesis pathways empowers synthetic biologists to integrate the acquired knowledge into heterologous systems, thereby enhancing the production of secondary metabolites in a cost-effective and tailored manner. As a result, RNA-seq studies hold particular significance, especially for non-model plants.
• This dataset represents the first instance of RNA-seq data encompassing the most critical tissue parts responsible for producing numerous medicinally important secondary metabolites. The sequencing was executed using the Novaseq platform, resulting in a read depth of 60 M in a single sample. Consequently, this dataset stands as a valuable resource, providing a foundation for subsequent gene expression analysis endeavors.
• Moreover, a sample induced with salicylic acid was also sequenced with the intention of facilitating future differential gene expression analysis. This specific setup offers an excellent opportunity to gain insights into how chemically induced plants elevate the production of secondary metabolites in *Datura metel* species.

## 2 Data Description

**Fig. 1:**
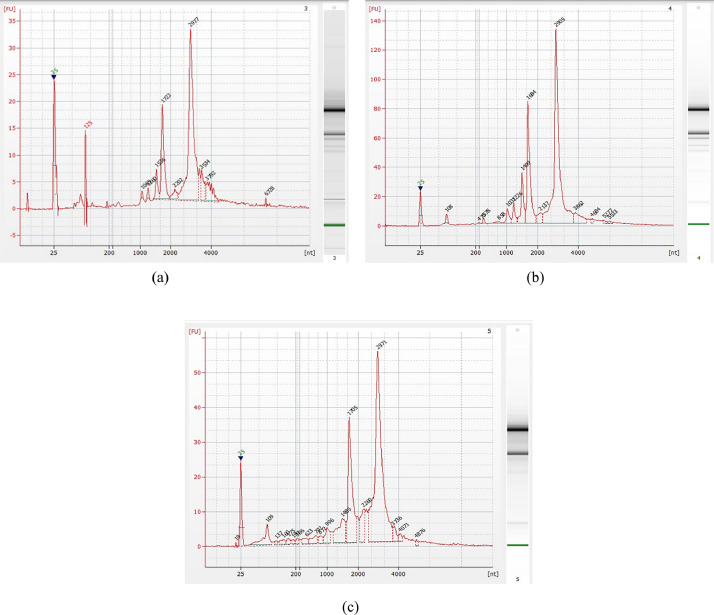
(a) Bioanalyzer electropherogram of total RNA for leaf tissue (b) Bioanalyzer electropherogram of total RNA for SA induced leaf (c) Bioanalyzer electropherogram of total RNA for flower tissue.

The Bioproject encompasses the raw sequencing data obtained through transcriptomic sequencing of leaf, salicylic-induced leaf, and flower tissues from *Datura metel* plants. This data was collected in January 2018 and has been deposited under the Bioproject with the accession number PRJNA838784. RNA was extracted from the leaf, salicylic-induced leaf, and flower tissues of *Datura metel* (Table 1 and Fig. 1) and used for *de novo* transcriptome analysis using Illumina NovaSeq 6000 sequencing technology. The 60x depth of sequencing coverage was received. The percentage of Q20 bases and Q30 bases were around 98 %, and 94 %, respectively (Table 1). The GC content was at least 45 % or above (Table 2).

**Table 1:** RNA concentration determined by NanoDrop.

Tissue Description	Concentration ng/µl	OD 260/280	OD 260/230
Leaf- 1	1204.4	2.10	2.25
Leaf – 2	1713.4	2.07	2.29
SA Induced Leaf-1	2814.7	2.12	2.32
SA Induced Leaf-2	1038.1	2.12	2.35
Flower-1	1250.1	2.11	2.28
Flower- 2	1374.3	2.06	2.26

**Table 2:** Raw RNA sequencing of the three tissues information.

Statistics	Total number of spots	Total number of bases	Total reads(bp)	GC percentage	Q20 %	Q30 %
DF_control	57,723,188	17,018,676,986	116,555,006	49.78	98.31	94.61
DL_treated	54,651,451	16,099,254,678	110,441,544	45.04	98.18	94.26
DL_control	57,710,549	17,026,416,161	116,747,642	47.67	98.06	93.99

**Fig. 2:**
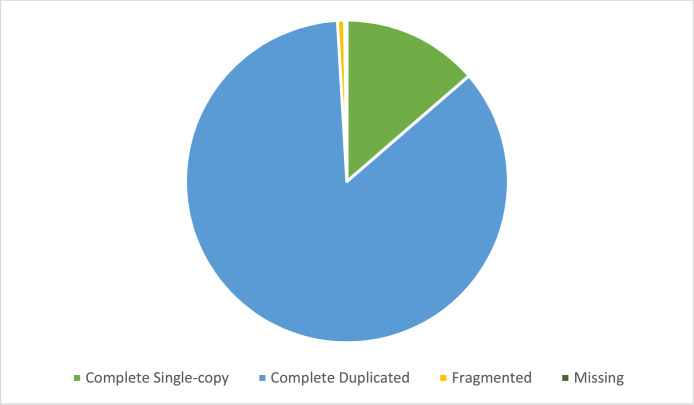
BUSCO analysis results. Blue represents the complete single-copy genes (85.41 %), green represents (13.65 %) of the complete duplicate-copy genes, and orange for fragmented genes (0.71 %) and missing genes (0.23 %). (For interpretation of the references to color in this figure legend, the reader is referred to the web version of this article.)

The sequencing results showed good quality for all tissues and provided valid information for subsequent analyses. *De novo* assembly of each transcriptome and a common transcriptome of leaf, induced leaf and flower buds were performed with Trinity 2.8.4 software. The default kmer size of 25 was set for the *de novo* transcriptome assembly of the flowers and leaves. The length of the assembled unigenes used for further study was ≥500 bp. All downstream analyses were based on high-quality clean data. All reads were *de novo* assembled into a single transcriptome individually and finally merged into a single transcriptome containing 232,032 contigs and representing 131,784 unigenes. The N50 was equal to 2967 bp for transcript 1866 bp for the longest isoform for the gene (Table 3). The mean lengths of transcripts and unigenes were 1529 bp and 978 bp, respectively. The contigs reported as the output of the Trinity package contain a FASTA file that contains redundancy. Therefore, the CD-HIT clusters [4] are used to remove duplicate sequences. The BUSCO completeness analysis [5] has revealed that 98 % of the completeness of the genome (Fig. 2).

**Table 3:** Transcriptome assembly metrics.

Length and other	Transcript
200–300	100
300–500	1250
500–1000	103,456
1000–2000	156,789
2000+	50
N50 Length	2967bp
Mean Length	1529bp

### 3.1 Bio project accession number: PRJNA838784 data deposition description

#### 3.1.1 Raw sequencing data for flower sample

• *Datura metel* flower: Illumina sequencing data were obtained from an Illumina NovaSeq 6000 run, producing 57.7 million spots with a total of 17 gigabases and 5 gigabytes of downloadable data. The experiment number is SRX17976631, and the corresponding raw data is labeled as Library ID “DF_control”. Other information is shown in Tables 4 and 5. The SRA accession number for the file is SRR21994161.
• *Datura metel* Salicylic Acid Induced Leaf: Illumina sequencing data were acquired from an Illumina NovaSeq 6000 run, yielding 54.7 million spots with a total of 16.1 gigabases and 4.8 gigabytes of downloadable data. The experimental number is SRX17976630, and the corresponding raw data is labeled as Library ID “DL_treated”. (Tables 4 and 5). The SRA accession number for the file is SRR21994162 and its statistical information is also provided in Tables 4 and 5.
• *Datura metel* normal Leaf: Illumina sequencing data were generated from an Illumina NovaSeq 6000 run, resulting in 57.7 million spots with a total of 17.1 gigabases and 5.1 gigabytes of downloadable data. The experimental number is SRX17976629, and the associated raw data is labeled as Library ID “DL_control**”** (Tables 4 and 5). The SRA accession number for the file is SRR21994163 and statistical information is given in Tables 4 and 5.

**Table 4:** Experimental description of the dataset.

SRA Accession Number	Title	File information	File Name	File type
SRR21994161	RNA_seq of *Datura metel*: flower	DF_control	clean_DF1_1.fastq.gz	fastq
			clean_DF1_2.fastq.gz	fastq
SRR21994162	RNA_seq of *Datura metel*: salicylic induced leaf	DL_treated	clean_DSL1_1.fastq.gz	fastq
			clean_DSL1_2.fastq.gz	fastq
SRR21994163	RNA_seq of *Datura metel*: leaf normal condition	DL_control	clean_DL1_1.fastq.gz	fastq
			clean_DL1_2.fastq.gz	fastq

**Table 5:** Experiment information.

Tissue type	Experiment	Library ID	Library Strategy	Library layout	Library Selection	Library layout	Platform	Instrument
Flower	SRX17976631	DF_control	RNA-seq	Transcriptomic	RT-PCR	Paired	Illumina	Illumina NovaSeq 6000
SA treated leaf	SRX17976630	DL_treated	RNA-seq	Transcriptomic	RT-PCR	Paired	Illumina	Illumina NovaSeq 6000
Normal leaf	SRX17976629	DL_control	RNA-seq	Transcriptomic	RT-PCR	Paired	Illumina	Illumina NovaSeq 6000

## 4 Experimental Design, Materials and Methods

### 4.1 Experimental design

In January 2018, *D. metel* seeds independently germinated in germination pots, with a 4:1 mixture of soil and sand. After 68 days of germination, the seedlings were transplanted into 2.5 × 2.0 m plots, spaced 50 × 60 cm apart, and cultivated within the greenhouse of the Sri Lanka Institute of Nano Technology.

### 4.2 RNA extraction

Previous investigations identified the optimal salicylic acid (SA) concentration for exogenous application as 5 mM for higher secondary metabolites production in Solanaceae plants [1]. As such, three plants had 10 ml of 5 mM SA sprayed on their third leaves every 12 h over a span of 36 h. Water was sprayed as the control for another three plants. Three hours following the final treatment, SA sprayed leaves and normal leaf and flower tissues were excised (two replicates from each tissue sample) and immersed in an RNA stabilization reagent, RNAlater (Cat 76104, Qiagen, Germany), to facilitate RNA isolation and subsequent transcriptome analysis. RNA extraction was executed using a Qiagen RNeasy plant mini kit, in adherence to the manufacturer's instructions. Quantitative and qualitative RNA assessment employed a NanoDrop-1000 spectrophotometer (Thermo Fisher Scientific Inc., Waltham, USA). The RNA purity was determined through absorbance measurements at 260 and 280 nm (A260/280) and 260/230 nm (A260/230), with ratios between 1.8 and 2.0 indicating optimal purity. RNA integrity was evaluated using an Agilent 2100 bioanalyzer, with a minimum RIN of 7.5 established as the threshold for our samples.

### 4.3 RNA sequencing

For RNA sequencing and library construction of *D. metel*, the RNA was dispatched to Macrogen in Korea. Trimmomatic software [2] was employed to filter raw reads, excluding sequences with adapters and low quality. Key parameters including N20, N30, N50 values, GC content, and clean data sequence duplication level were calculated. De novo assembly of individual transcriptomes and a shared transcriptome for leaf, induced leaf, and flower buds was executed using Trinity 2.8.4 software [3], with a default KMER size of 25 adopted for flower and leaf assemblies. Assembled unigenes exceeding a length of ≥500 bp were selected for further analysis, exclusively utilizing high-quality, clean data.

### 4.4 Quality control and sequence analysis

CD-HIT-EST (version 4.6.3) [4] analysis involved the selection of the longest isoform, and other contigs were merged based on a similarity criterion of 90 %. This step eliminated redundancy among FASTA sequences derived from the assembly. BUSCO [5] assessment evaluated the completeness of transcriptome assembly within the Viridiplantea (Kingdom) lineage, employing an E value cut-off of 1.03E-3.

## Limitations

Sample replicates were pooled and did not sequence separately.

## Ethics Statement

No human/animal data was used in this experiment.

The authors have read and followed the ethical requirements for publication in Data in Brief and confirmed that the current work does not involve human, or animal experiments or any data collected from social media platforms.

## CRediT authorship contribution statement

**Madhavi Hewadikaram:** Conceptualization, Methodology, Software, Data curation, Writing – original draft. **S.D.N.K. Bathige:** Supervision, Writing – review & editing. **Veranja Karunaratne:** Supervision, Writing – review & editing.

## Data Availability

Datura metel Raw sequence reads. (Original data) (NCBI SRA).
